# A systematic review of the burden of vaccine preventable pneumococcal disease in UK adults

**DOI:** 10.1186/s12890-016-0242-0

**Published:** 2016-05-11

**Authors:** James D. Chalmers, James Campling, Alison Dicker, Mark Woodhead, Harish Madhava

**Affiliations:** School of Medicine, University of Dundee, Dundee, DD1 9SY UK; Pfizer Ltd, Dorking Road, Tadworth, KT20 7NS UK; Manchester Royal Infirmary, Manchester, M13 9WL UK

**Keywords:** Vaccine, Pneumonia, Infection, Epidemiology, Mortality

## Abstract

**Background:**

Invasive pneumococcal disease (IPD) and pneumococcal pneumonia are common and carry a significant morbidity and mortality. Current strategies to prevent pneumococcal disease are under review in the United Kingdom (UK). We conducted a systematic review to evaluate the burden of vaccine type adult pneumococcal disease specifically in the UK.

**Methods:**

A systematic review conducted and reported according to MOOSE guidelines. Relevant studies from 1990 to 2015 were included. The primary outcome was the incidence of vaccine type pneumococcal disease, focussing on the pneumococcal polysaccharide vaccine (PPSV), the 13-valent conjugate vaccine (PCV13) and the 7-valent conjugate vaccine (PCV7).

**Results:**

Data from surveillance in England and Wales from 2013/14 shows an incidence of 6.85 per 100,000 population across all adult age groups for IPD, and an incidence of 20.58 per 100,000 population in those aged >65 years. The corresponding incidences for PCV13 serotype IPD were 1.4 per 100,000 and 3.72 per 100,000. The most recent available data for community-acquired pneumonia (CAP) including non-invasive disease showed an incidence of 20.6 per 100,000 for adult pneumococcal CAP and 8.6 per 100,000 population for PCV13 serotype CAP. Both IPD and CAP data sources in the UK suggest an ongoing herd protection effect from childhood PCV13 vaccination causing a reduction in the proportion of cases caused by PCV13 serotypes in adults. Despite this, applying the incidence rates to UK population estimates suggests more than 4000 patients annually will be hospitalised with PCV13 serotype CAP and more than 900 will be affected by IPD, although with a trend for these numbers to decrease over time.

There was limited recent data on serotype distribution in high risk groups such as those with chronic respiratory or cardiac disease and no data available for vaccine type (VT) CAP managed in the community where there is likely to be a considerable unmeasured burden.

**Conclusion:**

The most recent available data suggests that VT pneumococcal disease continues to have a high burden in UK adults despite the impact of childhood PCV13 vaccination. IPD estimates represent only a fraction of the total burden of pneumococcal disease.

**Study registration:**

PROSPERO CRD42015025043

**Electronic supplementary material:**

The online version of this article (doi:10.1186/s12890-016-0242-0) contains supplementary material, which is available to authorized users.

## Background

*Streptococcus pneumoniae* is a Gram-positive bacterium and a commensal of the human nasopharynx [1]. Failure of natural immunity to *S. pneumoniae* leads to pneumococcal infection and in some cases to invasive pneumococcal disease (IPD) [1–3]. The most frequent manifestation of pneumococcal disease, however, is pneumococcal pneumonia where the pneumococcus may be responsible for up to 60 % of cases of community-acquired pneumonia (CAP) [4]. Hospitalised CAP carries a mortality rate of 5–15 % rising to more than 30 % in patients admitted to the intensive care unit [5, 6]. The highest rates of pneumococcal disease are observed in infants, the elderly, patients with chronic respiratory disease and in patients with immune compromise [7–10]. This is despite the availability of effective antimicrobial treatments against *S. pneumonia*e, emphasising the importance of preventing pneumonia wherever possible [11, 12]. The impact of pneumococcal disease in the UK is substantial with approximately 6000 cases of IPD reported annually and 192,281 hospital admissions for pneumonia in 2013/14 in England of which up to 50 % may be pneumococcal [13, 14]. The cost to the UK National Health Service is estimated at more than £1 billion [15].

Pneumococcal disease is, at least partially, a vaccine preventable disease. The 23-valent pneumococcal polysaccharide vaccine (PPSV) has been recommended in the UK for patients at high risk of pneumococcal disease since 2003, including adults over the age of 65 years. A systematic review and meta-analysis of the data supporting PPSV show that it protects against IPD in adults in high income countries (OR 0.20 95 % CI 0.10–0.39, *n* = 27886), but limitations include uncertainty over its protection against IPD in patients with chronic illnesses (OR 1.56 95 % CI 0.35–0.694 *n* = 3230), protection against non-invasive pneumococcal CAP and its duration of protection [16].

The 13-valent pneumococcal conjugate vaccine (PCV13, Prevenar-13) has been evaluated for the prevention of vaccine type IPD in children and in elderly subjects [17, 18]. The recent CAPITA trial conducted in the Netherlands demonstrated the efficacy of PCV13 for the prevention, in those aged ≥65, of vaccine type pneumococcal CAP and also non-invasive CAP caused by vaccine serotypes [18]. An analysis based on the frequency of IPD and CAP caused by *S. pneumoniae* in the Netherlands concluded that PCV13 was cost-effective [19].

PCV13 is not currently part of the UK adult vaccination programme, neither for elderly patients aged >65 years nor for specific high risk groups. Determining whether PCV13 would be cost-effective in the UK requires accurate information on the burden of vaccine preventable pneumococcal disease in the UK.

We conducted a systematic review to determine the incidence and burden of vaccine preventable pneumococcal disease in the adult UK population.

## Methods

This manuscript reports a systematic review of observational studies and was conducted and is reported according to the MOOSE (meta-analysis and systematic review of observational studies in epidemiology) guidelines [20]. The review protocol was registered on PROSPERO (CRD42015025043).

### Search strategy

A librarian searched electronic databases from 1990 until September 2015 for relevant studies using PUBMED and EMBASE. A combination of text words and controlled vocabulary terms related to the subject of interest (pneumococcal disease) and possible outcome measures was used to develop a sensitive search strategy. Terms entered were (Streptococc* [tiab] OR pneumococc* [tiab]) AND (Serotype [Title/Abstract] OR serogroup [Title/Abstract) AND (incidence OR frequency OR prevalence OR distribution). Further searches were conducted for specific data on risk groups and UK regions as described in the relevant sections below. No language restrictions were applied to the search. The search was supplemented by reviews of reference lists, bibliographies and the investigators files where appropriate.

### Inclusion criteria

The review included observational cohort studies (including prospective, retrospective, registry and surveillance designs) reporting any of the following study outcomes; 1) Original data reporting of the incidence of vaccine type and non-vaccine type pneumococcal disease; 2) Inclusion or enrolment of patients in the United Kingdom; 3) Sufficient data to generate or infer incidence of disease in the general population or specific risk groups.

For data extraction, articles were independently reviewed by two investigators. Non relevant studies were excluded based on title and abstract review alone.

### Outcomes

The primary outcome was the incidence of vaccine type pneumococcal disease in the adult UK population, expressed as an incidence per 100,000 population. Secondary measures included the proportion of pneumococcal disease caused by PCV13 vaccine serotypes, other vaccine serotypes and the proportion of cases of CAP caused by vaccine serotypes was recorded. In addition the proportion of cases of vaccine type pneumococcal pneumonia in risk groups expressed as incidence per 100,000 population where possible. Risk groups include patients with splenectomy, chronic respiratory disease, chronic heart disease, chronic kidney disease and diabetes. Immunosuppression included any disorder leading to significant immune suppression (whether inherited or acquired) including HIV and iatrogenic immune suppression (incl. chemotherapy and corticosteroids).

### Vaccine type pneumococcal disease

Vaccine type (VT) pneumococcal disease was defined as being caused by one of the following serotypes: 4, 6B, 9 V, 14, 18C, 19 F, 23 F, 1, 3, 5, 6A, 7 F and 19A (PCV13 VT pneumococcal disease) OR pneumococcal disease caused by one of the following serotypes: 4, 6B, 9 V, 14, 18C, 19 F, 23 F, 1, 3, 5, 7 F, 19A, 2, 8, 9 N, 10A, 11A, 12 F, 15B, 17 F, 20, 22 F and 33 F. (PPSV VT pneumococcal disease).

Results were stratified according to the period of the study, following PPSV introduction (2003), following PCV7 introduction (2007) and following PCV13 introduction (2010).

## Results

**Fig. 1:**
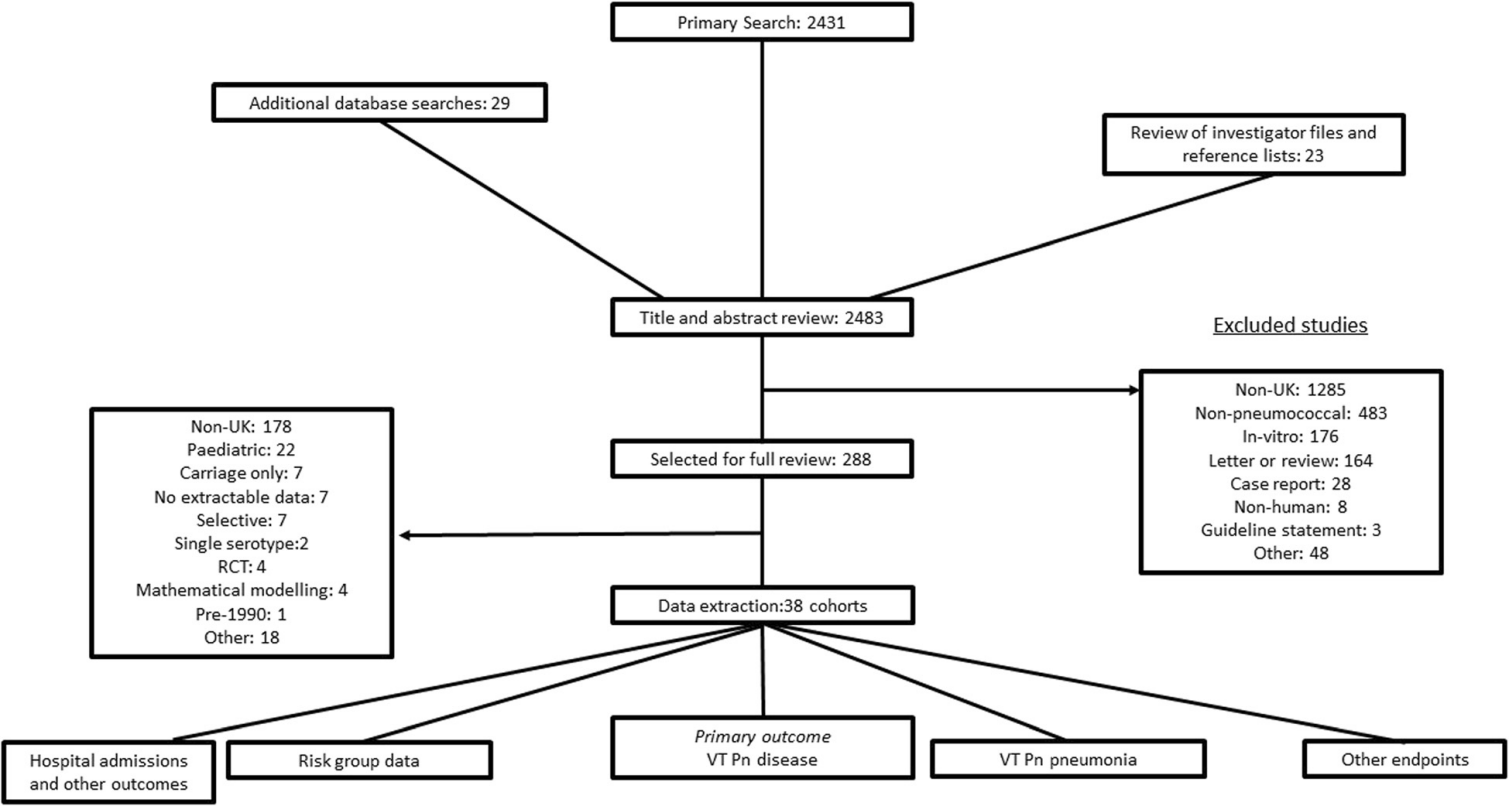
Process of literature review. Abbreviations UK = United Kingdom, RCT = randomized controlled trial, Pn = pneumococcal VT = vaccine type

The primary search identified 2,431 papers, with an additional 51 papers identified from other sources. 38 cohorts were eligible for inclusion. The characteristics of the included studies are described in the online supplement (Additional file 1: Tables E1-E5). The process of literature review is summarised in Fig. 1.

### Incidence of vaccine type pneumococcal disease in adults in the UK

The most recent published data for the incidence of VT pneumococcal disease is from 2013/14 in England and Wales, reported by Waight et al. [21] This data is limited to IPD.

Among all age groups (including children) PCV13 vaccine serotype IPD had an incidence of 1.40 per 100,000 population whilst PCV7 vaccine serotype IPD had an incidence of 0.2 per 100,000 population [21].

The same authors reported that this represents a statistically significant reduction in PCV13 vaccine serotype IPD compared to 2008–2010 (incidence 4.48 per 100,000 population), Incidence Rate Ratio (IRR) 0.31 (0.28–0.35) [21]. There was a corresponding increase in non-vaccine serotypes from 4.19 per 100,000 to 5.25 per 100,000 during the same period, IRR 1.25 (1.17–1.35). Regional data for the North East of England from 2006 to 2010 showed that PCV13 was responsible for 58 % of IPD cases across all age groups with a reported incidence of 7.8 per 100,000 falling to 5.2 per 100,000 in 2009/10. The corresponding figures for PCV7 serotypes were 3.9 per 100,000 in 2006/7 falling to 1.3 per 100,000 in 2009/10 [22].

The contribution of PCV13 serotypes to total IPD was relatively stable from 1996 to 2005, accounting for 76 % of cases in 1996, and 69 % in 2005 in a study from the Thames Valley region [23]. In the study of Waight et al., PCV13 serotypes accounted for 44.1 % of IPD across all age groups (42.4 % among adults aged 15 years and older) in 2008–10 falling to 20.4 % across all age groups (20.8 % among adults aged 15+) in 2013/14 [21].

Data from Scotland has also been reported [24–27]. Feikin et al. reported reductions in PCV7 serotypes following the introduction of the childhood vaccination schedule in 2007, with an IRR of 0.90 (0.61–1.35) in year one, 0.58 (0.38–0.88) in year 2, 0.29 (0.17–0.50) in year 3 and 0.16 (0.08–0.34) in year 4 post vaccine introduction [27]. These reductions were equivalent to those reported in other countries included in this analysis [27].

These data are limited to IPD. The search identified few studies that addressed non-invasive pneumococcal disease or that specifically addressed CAP. A series of studies conducted in Nottingham UK prospectively recruited patients admitted to hospital with CAP and used a validated multiplex immunoassay to determine 14 pneumococcal serotypes in urine [28–30]. The study of Rodrigo et al., which only included adults, found an incidence of PCV13 serotype CAP of 21.7 per 100,000 population in 2008/2009 reducing to 8.6 per 100,000 population in 2012–2013 [29]. The corresponding rates for PCV7 VT pneumococcal CAP was 11.1 per 100,000 in 2008/9 reducing to 2.3 per 100,000 in 2012–13 [29]. This was associated with a significant reduction in overall CAP from 90.7 cases per 100,000 in 2008/9 to 65.4 per 100,000 in 2012/13 [29]. This data is only applicable to hospitalised cases of CAP as outpatients were not included. Our systematic review identified no recent studies of the incidence of VT CAP managed in the community.

In terms of PPSV vaccine coverage of IPD, the proportion of pneumococcal disease cases caused by serotypes present in the vaccine, has remained stable over time. From 1995 to 1999, for those aged 5–64 years, 97.8 % of isolates were covered by the PPSV vaccine [31]. The study by Sleeman et al., identified slightly lower vaccine coverage (89.9 %) in Oxford whilst in those aged >65 years, vaccine coverage was 97.2 % [31]. Foster et al. reported coverage of 91 % for PPSV serotypes for invasive pneumococal disease in 1995 which remained stable at 89 % in 2005 [23]. During the similar period of 1993–1999 in Scotland, Kyaw et al. reported vaccine coverage of 95 % in adults age 5–64 years and 96 % in those older than 65 [32]. This remained stable over time, with 94.9 % coverage in 2003 from a Scottish study by Clarke et al. [33] Andrews et al. reported the incidence of IPD following the introduction of the PPSV programme to all adults in 2003 [34]. From 1998 to 2004/5 they report an incidence of 17.58 per 100,000 adults, with a stable incidence of 17.95 per 100,000 in 2005/6 and 17.2 in 2006–2010, among those aged 65–74 years [34]. They noted a small reduction in incidence of PPSV serotype IPD following the introduction of PCV7 but not following introduction of PPSV- IRR following PPSV in the over 80’s was 0.99 (0.90–1.08) while following introduction of PCV7 the IRR was 0.77 (0.71–0.83) [28]. The incidence rate remains substantial at 38.17 per 100,000 population in the over 80’s following PCV7 introduction [34]. Regional data confirms these patterns, with data from Hull and East Yorkshire (2002–2009) showing 89 % coverage for PPSV serotypes. This varied from 94.4 % in 2002 to 81.4 % in 2009, with the change arising entirely due to reductions in PCV7 serotypes [35].

### Burden of vaccine type pneumococcal community-acquired pneumonia

The majority of studies only reported data for IPD, and few specifically reported data for CAP. The only prospective study to report data on the contribution of VT pneumococcal CAP were from adults admitted to hospital in Nottingham, UK from 2008 to 2013 [29].

**Fig. 2:**
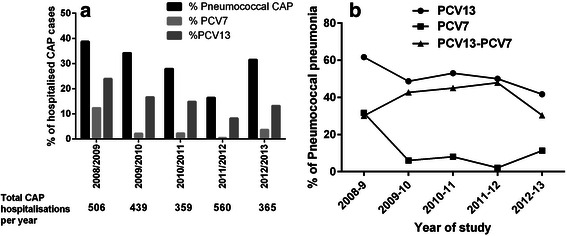
**a** Proportion of cases of adult hospitalised community-acquired pneumonia caused by *S.pneumoniae*, PCV7 serotypes and PCV13 serotypes respectively. **b** Proportion of pneumococcal CAP cases caused by different vaccine serotypes. Abbreviations. PCV = pneumococcal conjugate vaccine, CAP = community-acquired pneumonia

The proportion of cases of CAP caused by *S. pneumoniae* extracted from Rodrigo et al. [29] and the proportion of CAP cases caused by PCV7 and PCV13 are shown in Fig. 2.

**Fig. 3:**
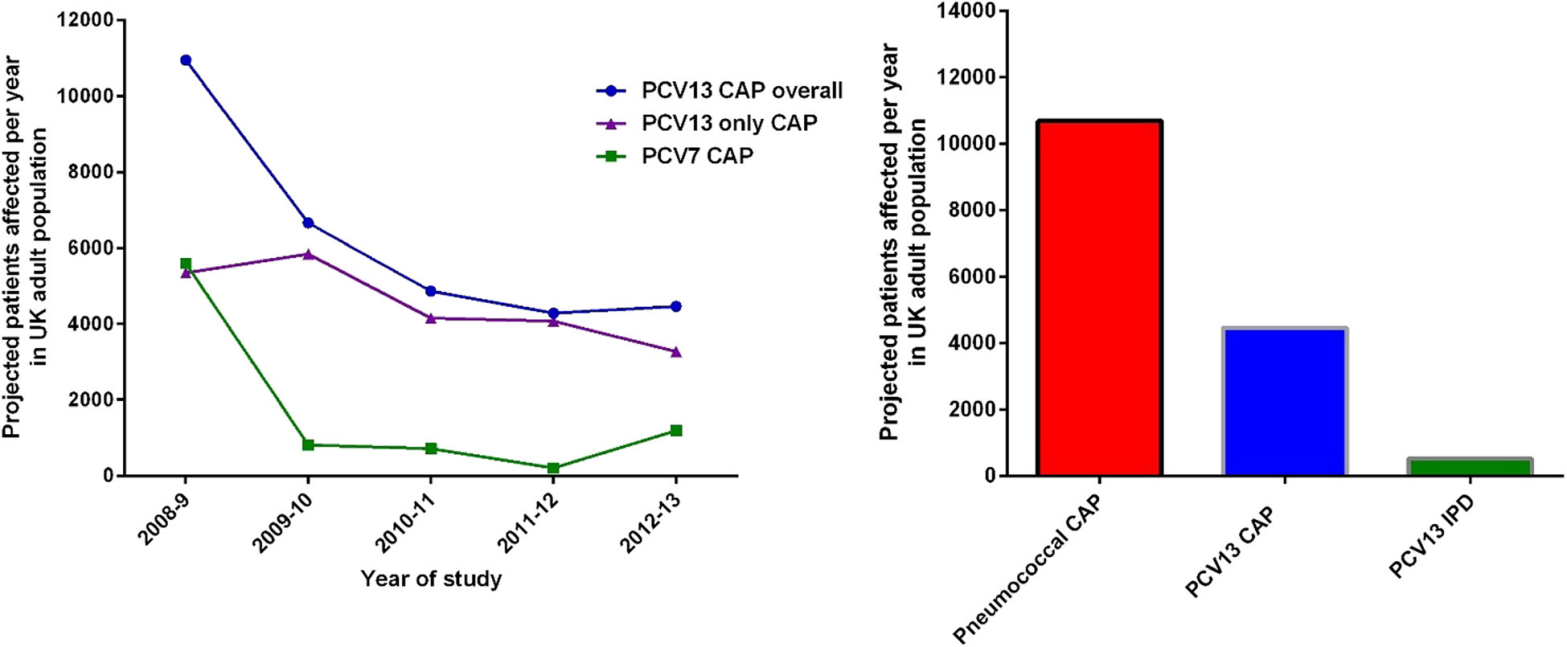
Projected number of patients affected by VT pneumococcal disease based on the most recent UK data identified in the systematic review [21, 29]. PCV13 only CAP refers to serotypes contained in PCV13 that are not contained in PCV7. Abbreviations: CAP = community-acquired pneumonia, IPD = invasive pneumococcal disease

This study reported a decline over 5 years in the incidence of all-cause CAP from 91 cases per 100,000 in 2008/9 falling to 65 cases per 100,000 in 2012/13. Pneumococcal pneumonia declined over the same period from 35 to 21 cases per 100,000 population. The proportion of cases of pneumococcal pneumonia caused by vaccine serotypes is shown below (Fig. 3). Overall, PCV13 serotypes accounted for 41–62 % of pneumococcal pneumonia during the study period, with the majority of these cases caused by the additional serotypes in PCV13 compared to PCV7 (namely 1,3,5,6A,7 F and 19A) [29]. This study found that only 13.3 % of pneumococcal aetiology was identified by blood cultures, consistent with comparison of population estimates which suggest the incidence of non-invasive CAP is 5 to 10 fold higher than IPD. [21, 29] Interestingly, although concerns have been raised that herd protection may not reduce the incidence of serotype 3, this study showed a marked reduction in serotype 3 CAP incidence from 2008 to 2013 [29].

The British Society for Antimicrobial Chemotherapy (BSAC) reported surveillance data from respiratory tract isolates of *S. pneumoniae*, although these represented sputum specimens without a clear clinical diagnosis of CAP [36]. Farrell et al. reported from 1997 to 2007 that PCV13 serotypes accounted for 58.6 % of respiratory tract isolates. The corresponding figure for PPSV was approximately 72.2 %, accounting for some serotypes not being reported as they were of low incidence [36].

### Pneumococcal meningitis

Limited data were also available for pneumococcal meningitis, with data from Johnson et al., showing that from 1998 to 2005, PCV13 serotypes accounted for 83.6 % of cases of pneumococcal meningitis in England and Wales [37]. PPSV serotypes accounted for 94 % of cases of pneumococcal meningitis [37].

### Absolute number of cases of PCV13 serotype CAP and IPD

The most recent Office for National Statistics estimates of the UK population are 54.3 m people in England, 64.5 m people across the whole of the UK, with 11.4 m people aged >65 years [38]. Putting the incidence data into context therefore, the most recent data would indicate that 934 cases of PCV13 serotype IPD would be expected in adults per year [21]. This would include approximately 420 cases in patients aged >65 years [21]. Assuming the results of Rodrigo et al. are applicable to the adult UK population as a whole, there would be 10,696 hospitalised cases of Pneumococcal CAP annually, with 4465 cases due to PCV13 serotypes [29]. This would include an estimated 2418 cases annually due to PCV13 serotypes in patients aged ≥65 years. There was no data to estimate the number of non-hospitalised (outpatient) cases due to vaccine serotypes. These estimates are shown in Fig. 3. The figure suggests that the true incidence from 2014/15 onwards is likely to be lower than reported above due to a decreasing trend in absolute numbers with time.

### Incidence of vaccine type pneumococcal disease in specific risk groups in the UK

#### Adults aged >65 years

Among adults aged >65 years Waight et al. reported an incidence of PCV13 serotype IPD of 10.33 per 100,000 population in 2008–10 reducing to 3.72 per 100,000 in 2013/14 [21]. PCV7 serotypes reduced from 4.58 per 100,000 in 2008–10 to 0.53 per 100,000 population in 2013/14, giving an IRR of 0.11 (0.08–0.18). This study demonstrated a highly significant reduction in all 5 additional serotypes while there were no significant reductions in the non-PCV13 serotypes [21].

From the study of Rodrigo et al., the overall contribution of pneumococcal CAP to overall CAP incidence varied from year to year from 17.1 to 37.3 % of cases [29]. The proportion of cases due to PCV7 reduced substantially from 13.4 % of all CAP cases in 2008/9 to 0.3 % in 2011/12. Rates of CAP due to PCV13 also reduced significantly from 2008/09 onwards, from 24.8 % of CAP cases to 7.5 % of cases in 2011/12 and 12.6 % of cases in 2012/13 [29]. The largest reductions were seen in those aged >85 years [29].

### Risk groups

Van Hoek reported data on the impact of clinical risk factors for IPD in England [39]. The authors examined specific risk groups including those with asplenia, chronic respiratory disease (including COPD), chronic heart disease, chronic renal disease, chronic liver disease, diabetes, immunosuppression, cochlear implants and cerebrospinal fluid leaks [39]. They used data from a 2009 survey of PPSV uptake in general practice to estimate the proportion of patients with these risk factors in England and identified 44.8 % of patients aged >65 years having had at least one risk factor, with chronic heart disease the most common. [39] The incidence of IPD was greatly increased in patients with risk factors, particularly chronic liver disease, immunosuppression and chronic respiratory disease. In the older age group (>65 years), the incidence in patients without risk factors was 17.9 per 100,000, increasing to 48 per 100,000 with one or more risk factor. This was higher still at 91 per 100,000 if the co-morbidity was COPD, and 129 per 100,000 in chronic liver disease [39].

A similar pattern was observed in younger adults (16–64 years). The baseline incidence without risk factors was 5.2 per 100,000, rising to 39 per 100,000 in risk groups, with the higher incidence 172 per 100,000 in those with chronic liver disease and 91 per 100,000 in chronic respiratory disease. The study was conducted prior to the introduction of PCV13. From 2005 to 2009, there was good coverage of PPSV in the risk groups (90 % in 2005/6 falling to 83 % in 2008/9, compared to 95 and 91 % in non-risk groups respectively), and also good coverage of PCV13 (73 % of IPD cases in 2005/6 falling to 61 % in 2008/9, compared to 75 and 64 % over the same period in non-risk groups) [39]. The introduction of PCV7 had a clear effect in both risk and non-risk groups, with the % vaccine coverage falling from 45 % in 2005/6 to 21 % in 2008/9, with no significant differences between risk and non-risk groups [39].

**Fig. 4:**
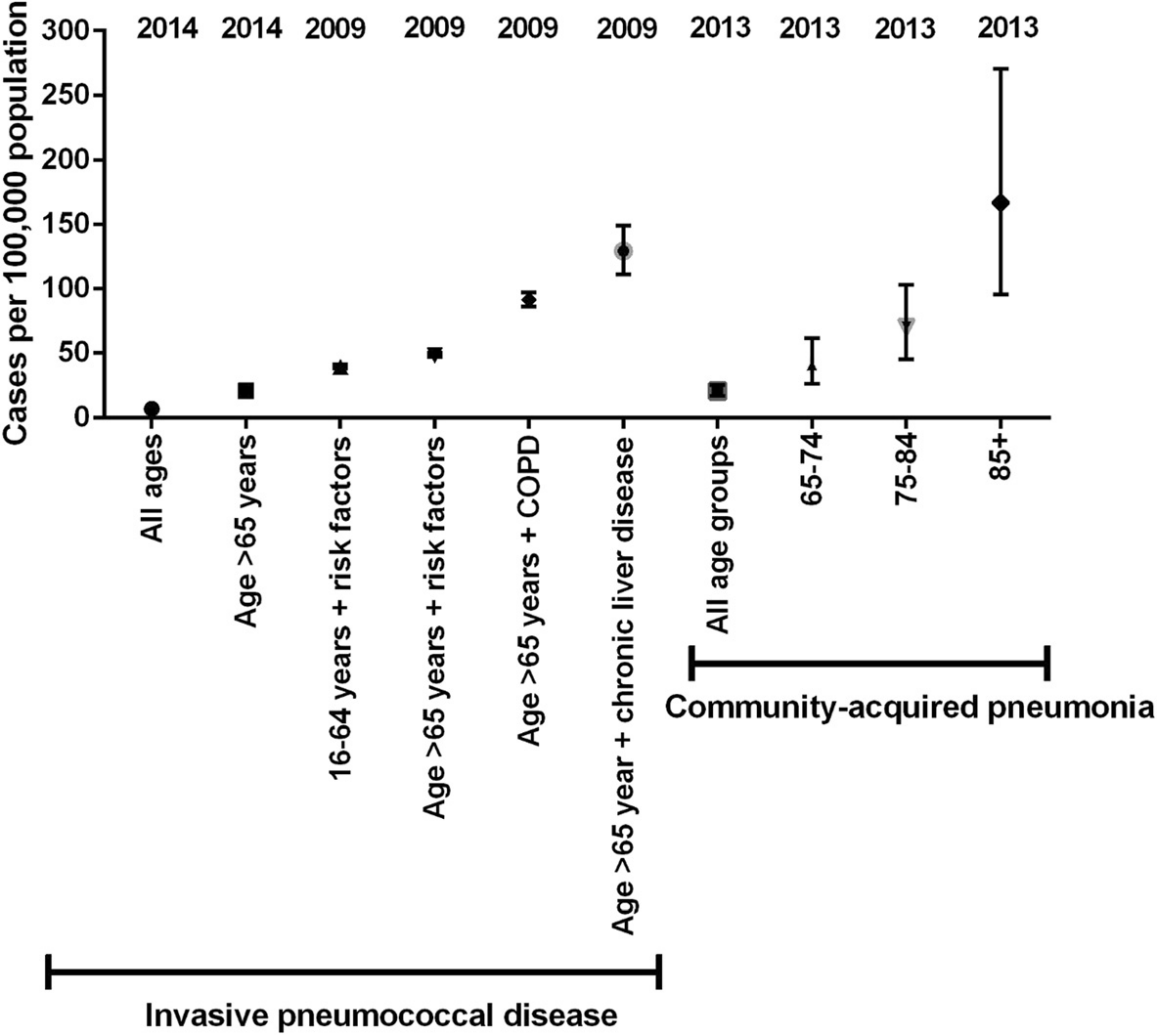
The most recent incidence data available for each pre-specified population grouped according to invasive pneumococcal disease and community-acquired pneumonia. Data derived from Waight et al., Rodrigo et al. and van Hoek et al. [21, 29, 39]. The dates across the top indicate the most recent year in which data were available

The search identified no specific data on the incidence of non-invasive pneumococcal infection in risk groups [10, 40–42]. A summary of the risk group incidence estimates are shown in Fig. 4. Table 1 below summarises incidence data from 3 studies with the most recent incidence data for IPD, CAP and risk groups in the UK.

**Table 1:** Selected population IPD and CAP disease burden estimates for PCV13 serotype CAP

Study	Population	Year	Incidence/100,000
Waight et al [21]	England and Wales IPD – all ages	2013/14	1.40
Waight et al [21]	England and Wales IPD- age >65 years	2013/14	3.72
Rodrigo et al [29]	Nottingham (multicentre) CAP- adults all ages	2012/13	8.60
Rodrigo et al [29]	Nottingham (multicentre) CAP- Age >65 years	2012/13	16.75
Van Hoek et al [39]	England Risk groups^a^ Adult	2008/9	37.10^b^
Van Hoek et al [39]	England Risk groups^a^ ≥65 years	2008/9	39.84^b^

CAP refers to non-invasive and invasive pneumococcal community acquired pneumonia

^a^risk groups include asplenia/splenic dysfunction/chronic respiratory disease/chronic heart disease/chronic kidney disease/chronic liver disease/diabetes/immunosuppression/cochlear implants/cerebrospinal fluid leaks [39]

^b^data extracted based on reported 83 % coverage of total IPD incidence by PCV13 during 2008/9

### Immunosuppressed patients

There are few studies of pneumococcal disease in immunosuppressed patients and most surveillance data are unable to identify these subgroups of patients. The above study by Van Hoek identified that immunosuppression was the single greatest risk factor for IPD among risk groups in the UK population [39]. The incidence was 209 per 100,000 for immunosuppressed patients aged >65 years (odds ratio 11.7 compared to patients >65 without immunosuppression). The equivalent data for younger adults was 88 per 100,000 (odds ratio 17.1 compared to adults 16–64 without immunosuppression [39]. HIV is also a risk factor for IPD. [42] The above study identified an incidence of 95 per 100,000 in the elderly (age >65 years) although this is based on only 2 cases. The incidence rate was 316 per 100,000 in adults aged 16–64 years (odds ratio 61.2 compared to patients without HIV) [39].

Yin et al. reported a study of IPD among HIV positive individuals from 2000 to 2009. This included 63,109 HIV positive adults of which 951 developed IPD. [43] This resulted in estimates of IPD incidence of 245 per 100,000 HIV positive adults. The study reported that in the final year of data (2009), 23 % of causative serotypes were covered by PCV7, a 54 % reduction compared to prior to PCV7 introduction. For PPSV, the coverage was 89 % in 2000–2006 and 91 % in 2009 [43].

### Impact of vaccine type pneumococcal disease on outcomes

We identified minimal data on the pre-specified markers of disease impact such as hospitalisation rates, length of hospital stay, intensive care unit admissions, attributable mortality and healthcare costs.

Van Hoek examined differences in site of infection and mortality association with different vaccine serotypes in England and Wales. Serotypes 35 F, 6C and 18C were most frequently associated with meningitis in the elderly, and serotype 1 was most strongly associated with empyema hospitalisation [44].

The highest case fatality rates among patients aged 5–64 years were reported for serotype 31 (33 %), 11A (30 %) and 19 F (21 %). Serotype 31 is not included in either the PPSV or PCV13, while 11A is included in PPSV and not PCV13. Among the elderly (>65 years) the highest case fatality rates were for serotypes 19 F (41 %), 31 (40 %) and 3 (39 %) [44].

Scottish data was reported by Inverarity et al. for the period 1992–2007. The highest 30-day mortality rate was for serogroup 3 (24 %), followed by 19 and 23 (18 and 15 % respectively). Serotype data was not available for the majority of the study [26].

Risk groups greatly influence the risk of mortality in IPD. As reported by Van Hoek, the mortality in patients aged >65 years without other risk factors was 29.1 % (compared to 5.4 % in patients aged 16–64 years) [39]. Among the elderly, one or more risk factors increased mortality by approximately 20 %, chronic heart disease increased risk by 40 %, kidney disease by 90 % and chronic liver disease was associated with a near 3 fold increased risk of death [39]. Even larger impacts were seen in the younger age group, where having one or more co-morbidity increased mortality by an odds ratio of 3.9 (3.4–4.4) [39].

## Discussion

Our systematic review identifies a high burden of pneumococcal disease in adults in the UK, while also revealing substantial ongoing changes in the epidemiology of pneumococcal disease. The most recent data from 2013/14 shows an incidence of 6.85 per 100,000 population across all age groups for IPD, and an incidence of 20.58 per 100,000 population in those aged >65 years [21]. The corresponding incidences for PCV13 serotype IPD were 1.4 per 100,000 and 3.72 per 100,000. The most recent available data for CAP including non-invasive disease showed an incidence of 20.6 per 100,000 for hospitalised adults with pneumococcal CAP and 8.6 per 100,000 population for hospitalised PCV13 serotype CAP [21, 29]. We have estimated that if these most recent estimates are applicable to UK population as a whole, there would be at least 10,000 cases of hospitalised pneumococcal CAP, with 4000 caused by PCV13 serotypes and more than 900 cases of PCV13 serotype IPD. The estimates of burden for non-invasive disease are likely to be an underestimate due to the absence of data from CAP managed in the community.

These data have limitations as discussed below, but suggest that pneumococcal disease and PCV13 vaccine type pneumococcal CAP continue to have a significant burden in adults, even after the introduction of PCV13 in children.

There is, however, an ongoing trend of reduced incidence of PCV13 serotype IPD and CAP in the UK, demonstrated both in the study of Waight et al., who demonstrated a 69 % incidence reduction between 2008 and 2014, and in the study by Rodrigo et al. who demonstrated a reduction of 48 % in PCV7 CAP, and a 13 % reduction in the additional serotypes contained in PCV13 [21, 29].

Pneumococcal vaccination is now a core part of public health policy [3, 45]. The pneumococcal polysaccharide vaccination covers 23 common serotypes and has been available since 1983. It was introduced into the routine vaccination schedule in England and Wales in 2003/4 for patients aged 80 and over, followed by patients aged 75 and over in 2004/5 and all patients aged 65 and over in 2005/6. The 7-valent conjugate vaccine was introduced in children in 2007, followed by the introduction of the 13-valent conjugate vaccine for children in 2010 [3].

*S. pneumoniae* is capable of causing IPD and non-invasive pneumococcal pneumonia [46]. We identified a large body of evidence on the incidence of IPD in the UK thanks to ongoing surveillance programmes in England, Wales and Scotland.

Patients aged >65 years have the highest incidence of pneumococcal disease and non-invasive pneumococcal pneumonia and are therefore the primary target of vaccination programmes [18]. Although high uptake of paediatric conjugate vaccines has led to a reduction in cases of adult IPD through herd protection, there remains a substantial burden of IPD and pneumococcal pneumonia in adults [47, 48].

The CAPITA trial was a randomized double blind placebo controlled trial conducted in the Netherlands which demonstrated efficacy in the reduction of vaccine type pneumococcal CAP in those adults >65 years receiving the 13-valent conjugate vaccine [18]. In the per-protocol population, vaccine efficacy for the prevention of a first episode of vaccine-type CAP was 46 %, and protection persisted for at least 4 years. There are, however, important differences between the UK and the Netherlands. The Netherlands has no PPSV programme, while uptake of the PPSV vaccination in the UK is among the best in Europe [45]. In addition, as noted above, the PCV13 vaccine has been used in the UK since 2010 for children, while the CAPITA trial results were obtained in a population where PCV7 was introduced for newborns in 2006 and replaced by PCV10 in 2011 [49]. Therefore our data allows a degree of comparison between the epidemiology of pneumococcal disease in the UK and the Netherlands. In 2008 in the Netherlands, 68.4 % of IPD episodes in patients aged 65 years or older were caused by PCV13 serotypes, compared to 42.3 % in 2013 [49]. The corresponding figures from Waight et al. for England and Wales were 44.1 and 20.3 % [21]. Rodrigo et al. reported that 40.6 % of cases of pneumococcal CAP were PCV13 VT in 2012/13 [29].

The impact of pneumococcal vaccination programmes in children has been accompanied by a concern about serotype replacement and the potential implication of this on public health [50–52]. Waight et al. reported a significant increase in non-vaccine serotype IPD following the introduction of PCV13 (IRR 1.25 95 % CI 1.17–1.35 comparing 2008–10 to 2013/14) [21]. Data reported, only in abstract form to date, from the BSAC surveillance project provides further evidence that this is occurring [53]. Using data from up to 40 clinical laboratories in the UK and Ireland, they showed for bacteraemic pneumococcal disease, 76 % of cases were PCV13 serotypes prior to PCV7 introduction (data from Jan 04- Dec 06) falling to 21 % (Jan-Dec 14). The corresponding data for respiratory isolates was 59 % falling to 17 % [53]. The most frequent serotypes for both bacteraemia and lower respiratory tract infection in 2004–6 were covered by PCV13, but in 2014 were not, with serotypes 8, 22 F and 12 F being most frequent in bacteraemia and 15A and 23B most frequent in LRTI [53]. They report associated significant increases in penicillin, tetracycline and multidrug resistance which appears to be mostly due to expansion of serotype 15A [53]. Antimicrobial resistance is an increasing problem in CAP generally and these data emphasise the importance of ongoing surveillance and consideration of the indirect impact of childhood vaccination [50–52, 54].

This study identified important gaps in the literature regarding the burden of pneumococcal disease in the UK. There was limited data outside of surveillance of IPD. Data for non-invasive pneumococcal pneumonia was limited to hospitalised patients in a single UK city, in Nottingham, England and a series of respiratory tract isolates forming part of the British Society for Antimicrobial Chemotherapy surveillance studies [31, 36, 53] which would include patients with CAP, but would also include respiratory/sputum isolates from patients with other respiratory tract infections such as exacerbations of COPD [55], bronchiectasis (where *S. pneumoniae* can be a coloniser) [56] and upper airway commensals. There are important differences both in terms of serotypes responsible for invasive vs non-invasive disease, in disease outcomes and in vaccine effectiveness against non-invasive disease vs invasive disease. Inclusion of additional data on non-invasive CAP in the UK would be valuable. Our available data suggests that there are up to 10 cases of non-invasive pneumococcal CAP for each case of IPD and so studies restricted to IPD are likely to greatly underestimate the burden of pneumococcal disease.

None of the studies identified in the systematic review were conducted in the community and so the burden of pneumococcal disease in the community is unknown. Multiple studies suggest that *S. pneumoniae* remains the most common cause of CAP in outpatients, where the majority of CAP is managed [57–60]. Although the mortality rate of CAP in the community is low at less than 1 %, the impact is significant at a population level in terms of lost days of work, reduced productivity and long term complications [60, 61]. More data on the burden of disease in the community is needed, as hospital based studies may underestimate the true impact.

At the other end of the severity spectrum, we identified little data on the pneumococcal vaccine serotypes associated with the most severe pneumonia causing intensive care unit admission. Although Van Hoek et al. reported data on the mortality attributable to different serotypes using record linkage, similar data for ICU admission was not available [39]. Mortality and severe pneumonia requiring ICU admission are not necessarily synonymous as the majority of pneumonia related deaths occur outside the ICU with up to 50 % occurring due to co-morbidities rather than directly due to pneumonia [61–63].

The incidence of CAP, and pneumococcal disease in particular is greatly increased in at risk populations such as patients with COPD, chronic heart failure and immunosuppression [42]. COPD patients have a greatly increased frequency of pneumonia at 22.4 per 1000 person years due to a combination of reduced local immunity, co-morbidities and the impact of immunosuppression through inhaled corticosteroids [64, 65]. Additional risk factors identified through analysis of UK health records include diabetes, chronic heart disease, chronic renal disease, asplenia, chronic liver disease, sickle cell disease, HIV, previous stroke, rheumatoid arthritis, Parkinson’s disease, multiple sclerosis, dementia, osteoporosis and malignancy [66]. IPD data suggests a greatly increased risk of IPD in patients with one or more risk factors, particularly chronic liver disease, COPD and immunosuppression with evidence of “risk stacking” with the incidences greatest in patients with more risk factors, or a combination of increasing age and high risk co-morbidities [39]. Mortality from IPD is also greatly increased in patients from high risk groups, ranging from a 20 % increase in risk of death among elderly patients with COPD, to an estimated 1000 % increased risk of death among patients aged 16–64 years with chronic liver disease, compared to patients of the same age without liver disease [39].

The UK population is ageing, and therefore the burden of CAP and pneumococcal disease in general is expected to increase even with the impact of childhood and adult vaccine programmes.

There are important regional variations in the incidence of CAP in the UK. Millet et al. demonstrated a significantly lower incidence of LRTI and CAP in London and the South East of England compared to the North, Yorkshire and the Midlands in both men and women [67]. This may be partly explained by differences in socioeconomic deprivation which is a major risk factor for CAP. Given the large differences observed in this study between regions, estimates of risk averaged across the whole of the UK should be interpreted in the local context. [67]

## Conclusion

VT pneumococcal disease continues to have a significant burden in adults in the UK. IPD data will underestimate the impact of pneumococcal disease because the majority of cases of CAP are non-invasive. Nevertheless, both IPD and CAP data sources in the UK suggest an ongoing herd protection effect from childhood PCV13 causing a reduction in the proportion of cases caused by PCV13 serotypes in adults. Despite this, the most recent data suggests that PCV13 serotypes account for 12.6 % of all cases of CAP and 41 % of pneumococcal CAP. This data will be useful in evaluating the clinical and economic case for adult PCV13 vaccination in the UK.

### Ethical approval

Not required.

### Availability of data

Data available from the authors on request.

## Additional file

Additional file 1: Table of included studies. (DOCX 26 kb)

## Abbreviations

BSAC: British Society of Anti-microbial chemotherapy
CAP: community acquired pneumonia
COPD: chronic obstructive pulmonary disease
HIV: human immunodeficiency virus
IPD: invasive pneumococcal disease
IRR: incident rate ratio
LRTI: lower respiratory tract infection
MOOSE: meta analysis of observational studies of epidemiology
NHS: National Health Service
PCV13: pneumococcal conjugate vaccine- 13-valent
PCV7: pneumococcal conjugate vaccine- 7-valent
PPSV: pneumococcal polysaccharide vaccine
RCT: randomized controlled trial
UK: United Kingdom
VT: vaccine type
