# Supplementary material for: A systematic review of the burden of vaccine preventable pneumococcal disease in UK adults
Source: BMC Pulm Med. 2016 May 11;16:77. doi: 10.1186/s12890-016-0242-0 (PMC4864929; doi:10.1186/s12890-016-0242-0)
Supplement: Additional file 1: — Table of included studies. (DOCX 26 kb) [file 12890_2016_242_MOESM1_ESM.docx]

| **First author** | **year of publication** | **Location** | **included patients** | **years of enrolment** | **Study design** | **included population** | **method of serotype determination** | **Number of patients with Pneumococcal disease** | **total number of adult patients with pneumonia** | **Study conclusion** |
| --- | --- | --- | --- | --- | --- | --- | --- | --- | --- | --- |
| Johnson (1) | 1996 | England and Wales | 1990 and 1995 | retrospective surveillance | 1127 individual patient isolates | Laboratory isolates | not described | 1127 | Laboratory isolates only | Increasing resistance to penicillin and erythromycin |
| Sleeman (2) | 2001 | England and Wales | invasive pneumococcal disease, all ages | 1995-1997 | retrospective-surveillance | Not described | Quellung reaction | 4562 | not reported | PCV7 serotypes cause 66% of IPD in adults >65yrs |
| Johnson (3) | 2007 | England | all (adult population is 15+) | 1998-2005 | Hospital episode statistics | pneumococcal meningitis only | ELISA | 1978 | 0 | PCV7 coverage of 20-50% for adults>65yrs |
| Pichon (4) | 2009 | England and Wales | aged >80 years | 2003 and 2004- winter only | retrospective, selection of laboratory isolates | Invasive pneumococcal disease | Agglutination method | 542 | not reported | Limited evidence of serotype switching |
| Trotter (5) | 2010 | England and Wales | all ages adults and children, invasive pneumococcal disease | 1996-2006 | retrospective surveillance | Invasive pneumococcal disease | anti-sera | 52,579 | not reported | Baseline serotype data prior to introduction of PCV7 |
| Miller (6) | 2011 | England and Wales | all ages | 2000-2010 | retrospective surveillance | invasive pneumococcal disease | not reported | not specifically reported | not reported | Early evidence of herd effects in older people with PCV7 |
| van Hoek (7) | 2012 | England and Wales | all ages | 2002-2011 | retrospective surveillance with record linkage | invasive pneumococcal disease | antisera | 23688 | not reported | Serotypes have different presentations and outcomes |
| Andrews (8) | 2012 | England and Wales | Age >65 | 1998-2010 | retrospective surveillance | invasive pneumococcal disease | not reported | 31048 | not reported | PPV23 remains cost effective in the UK |
| Pichon (9) | 2013 | England and Wales | all ages adults and children, | 2004-2009 | retrospective surveillance | Pneumococcal meningitis | various methods | 1030 | 0 | Increase in non-PCV7 serotypes following PCV7 introduction |
| Waight (10) | 2015 | England and Wales | all ages | 2000-2014 | retrospective-surveillance | Invasive pneumococcal disease | not reported in that format | not reported as total numbers | Not reported | Herd effects are eliminating vaccine serotypes |

Table E1. Studies conducted in England and Wales. Studies are listed in order of publication.

| **First author** | **year of publication** | **Location** | **included patients** | **years of enrolment** | **Method** | **included population** | **method of serotype determination** | **Number of patients with Pneumococcal disease** | **total number of adult patients with pneumonia** | **Study conclusion** |
| --- | --- | --- | --- | --- | --- | --- | --- | --- | --- | --- |
| Urwin (11) | 1996 | North East Thames | All age groups | 1991-1993 | Prospective laboratory surveillance | Pneumococcal meningitis | Slide agglutination | 114 | meningitis | 90% of serotypes covered by PPV23 |
| Balakrishnan (12) | 2000 | London, England | Adults and children | 1992-1998 | Restrospective surveillance | Invasive pneumococcal disease | Not described | 107 | Not reported | Described risk factors for fatal outcome |
| Sleeman (2) | 2001 | Oxfordshire, England | invasive pneumococcal disease, all ages | 1995-1999 | retrospective-surveillance | Invasive pneumococcal disease | Quellung reaction | 1297 | not reported | As described above (subgroup in one region) |
| Birtles (13) | 2004 | Reading, England | invasive pneumococcal disease, all ages | 2000-2001 | retrospective of lab isolates | adults and children | anti-sera | 56 | not reported | Description of sequence types in a single hospital |
| Leeming (14) | 2005 | South-West England | pneumonia | 1999-2002 | prospective observational study | adults >16 years with pneumonia and invasive pneumococcal disease | serotype specific ELISA in urine | 86 | 64 | Validation of the serotype specific urinary antigen detection assay |
| Sheppard (15) | 2007 | London, England | Aged <50 years with CAP requiring ICU or HDU admission or a patient with CAP felt to be unusual | 2003 | Retrospective, outbreak | Community-acquired pneumonia | ELISA | 11 | 11 | Utility of serotyping in the context of an outbreak |
| Ihekweazu (16) | 2008 | South West England | all ages | 1996-2005 | retrospective surveillance | Invasive pneumococcal disease | "standard methods" | 5693 | not reported | Epidemiology of IPD prior to vaccine introduction. |
| Foster (17) | 2008 | Oxfordshire, England | unselected adults and children | 1996-2005 | retrospective, surveillance | Invasive pneumococcal disease | Quellung reaction | 2691 | not reported | Epidemiology of IPD prior to vaccine introduction |
| Elston (18) | 2012 | Hull and East Yorkshire, England | all ages | 2002-2009 | not reported | invasive pneumococcal disease (data on overall pneumonia admissions reported) |  | 653 (IPD) | Not reported | Limited impact of existing vaccine programmes on pneumococcal disease |
| Chapman (19) | 2012 | North East England | all ages | 2006-2010 | retrospective surveillance | invasive pneumococcal disease | not reported | 1088 | not reported | Evidence of PCV7 serotype replacement |
| Bewick (20) | 2012 | Nottingham | age >16 years and diagnosed with community-acquired pneumonia | 2008-2010 | prospective observational study | community-acquired pneumonia | Biorad multiplex immunoassay | 366 | 366 | Describes serotype prevalence in Single UK region |
| Rodrigo (21) | 2014 | Nottingham | Age >16 years and diagnosed with community-acquired pneumonia | 2008-2011 | prospective observational study | community-acquired pneumonia | Biorad multiplex immunoassay | 410 | 410 | Child contact and child vaccine status impact on pneumococcal susceptibility |
| Rodrigo (22) | 2014 | Nottingham | Age >16 years and diagnosed with community-acquired pneumonia | 2008-2011 | prospective observational study | 1166 patients with community-acquired pneumonia (invasive and non-invasive) | Biorad multiplex immunoassay | 415 | 415 | Characteristics of patients with PCV7 serotype pneumonia |
| Rodrigo (23) | 2015 | Nottingham | Age >16 years and diagnosed with community-acquired pneumonia | 2008-2011 | prospective observational study | community-acquired pneumonia | Biorad multiplex immunoassay | 653 | 653 | Evidence of PCV13 herd effects in adult pneumococcal pneumonia |

Table E2, Regional and single centres studies.

| **First author** | **year of publication** | **Location** | **included patients** | **years of enrolment** | **Method** | **included patients** | **method of serotype determination** | **Number of patients with Pneumococcal disease** | **total number of adult patients with pneumonia** | **Study conclusion** |
| --- | --- | --- | --- | --- | --- | --- | --- | --- | --- | --- |
| Kyaw (24) | 2000 | Scotland | invasive pneumococcal disease, all ages | 1993-1999 | retrospective surveillance | invasive pneumococcal disease | Not reported | 5659 | Not reported | >95% coverage with PPV23 |
| McKenzie (25) | 2000 | Grampian, Scotland | bacteraemia | 1993-1995 | retrospective - surveillance | adults and children with pneumococcal bacteraemia | co-agglutination | 103 | not reported | Emergence of an erythromycin resistant clone |
| Kyaw (26) | 2002 | Scotland | non-invasive pneumococcal disease | 1988-1999 | retrospective study of non-invasive isolates | adults and children admitted to hospital with acute respiratory infection | co-agglutination | 4491 | not clear, 4491 with acute respiratory infection but not specifically pneumonia | 74-94% of serotypes covered by PCV 7 to PCV 11 vaccines. |
| Denham (27) | 2004 | Scotland | invasive pneumococcal disease, all ages | 1999-2002 | retrospective surveillance | invasive pneumococcal disease | co-agglutination | 1741 | not reported | Establishes baseline serotypes prior to vaccine introduction |
| Clarke (28) | 2004 | Scotland | invasive pneumococcal disease, all ages | 2003 | retrospective surveillance | invasive pneumococcal disease | co-agglutination | 367 | not reported | 94.9% PPV23 coverage and 50-64% conjugate vaccine coverage |
| Mooney (29) | 2008 | Scotland | All age groups | 2003/2004 winter | Retrospective cohort study | Invasive pneumococcal disease | Not described | 442 | Not reported | PPV23 introduction was associated with a reduction of 1/3 in the IPD incidence in the elderly |
| Jefferies (30) | 2010 | Scotland | All ages | 2001-2006 | retrospective, surveillance | Invasive pneumococcal disease | coagglutination | 2838 | Not reported | Changes in serotypes prior to childhood vaccination |
| Inverarity (31) | 2011 | Scotland | all ages | 1992-2007 | retrospective surveillance | invasive pneumococcal disease | co-agglutination | 5959 | not reported | Risk of death from IPD varies by serotype |
| Lamb (32) | 2014 | Scotland | all ages | 1999-2010 | retrospective surveillance | invasive pneumococcal disease | co-agglutination | not reported | invasive pneumococcal disease | 2014 |

Table E3. Scottish studies.

| **First author** | **year of publication** | **Location** | **included patients** | **years of enrolment** | **Method** | **included patients** | **method of serotype determination** | **Number of patients with Pneumococcal disease** | **total number of adult patients with pneumonia** | **Study conclusion** |
| --- | --- | --- | --- | --- | --- | --- | --- | --- | --- | --- |
| Henriques (33) | 2000 | Canada, UK, Spain, Sweden, USA | pneumococcal bacteraemia, aged >18 | 1993-1995 | prospective observational study | Invasive pneumococcal disease | gel diffusion | 354 | not reported | Only included 9 from UK |
| Farrell (34) | 2008 | UK and Ireland | Invasive and non-invasive pneumococcal disease | 2005-6* (*Extractable UK data)) | retrospective surveillance | adults and children with bacteraemia or respiratory isolation (predominantly sputum) | 2001-2004= slide agglutination test, 2005 onwards, ELISA | 354 bacteraemia, 749 respiratory | bacteraemia or respiratory isolates which may be pneumonia or another respiratory infection e.g exacerbation of copd | Prior to PCV7 introduction, good coverage of PCV7 serotypes. Low frequency of antibiotic resistance. |
| Feikin (35) | 2013 | Worldwide- includes data from England and Wales and Scotland | all ages | 5 years before and up to 10 years after introduction of PCV7 in individual countries | retrospective surveillance | invasive pneumococcal disease | various methods | -international | Interational | Evidence of herd effect from PCV7 childhood vaccination |
| Torne (36) | 2014 | Reported as UK, but likely to be England only | all ages | 2010 | retrospective surveillance | invasive pneumococcal disease | not reported | not reported | invasive pneumococcal disease | European coverage of PV10 and PCV13 were 46.1% and 73.1% |

Table E4. International studies or miscellaneous studies.

| **First author** | **year of publication** | **Location** | **included patients** | **years of enrolment** | **Method** | **included patients** | **method of serotype determination** | **Number of patients with Pneumococcal disease** | **total number of adult patients with pneumonia** | **Study conclusion** |
| --- | --- | --- | --- | --- | --- | --- | --- | --- | --- | --- |
| Yin (37) | 2012 | England and Wales | Adults >15 years and HIV positive | 2000-2009 | Retrospective data linkage study | Invasive pneumococcal disease | Not reported | 951 | Not reported | Evidence of herd effect on IPD extends to patients with HIV |
| Van Hoek (38) | 2012 | England | Adults and children | 2002-2009 | Retrospective record linkage | Invasive pneumococcal disease | Not reported | 38,055 (22,298 linked to hospital episode statistics) | Not reported | A marked increase in IPD among certain risk groups |

Table E5. Studies of specific risk groups

REFERENCES

1. Johnson AP, Speller DCE, George RC et al. Prevalence of antibiotic resistance and serotypes in pneumococci in England and Wales: results of the observational surveys in 1990 and 1995. *BMJ* 1996;312:1454-6.
2. Sleeman K, Knox K, George R *et al*. Invasive pneumococcal disease in England and Wales: vaccination implications. *J Infect Dis* 2001; **183**: 239-46.
3. Johnson AP, Waight P, Andrews N *et al*. Morbidity and mortality of pneumococcal meningitis and serotypes of causative strains prior to introduction of the 7-valent conjugant pneumococcal vaccine in England. *J Infect* 2007; **55**: 394-9.
4. Pichon B, Bennett HV, Efstratiou A et al. Genetic characteristics of pneumococcal disease in elderly patients before introducing the pneumococcal conjugate vaccine. *Epidemiol Infect* 2009;137(7):1049-56.
5. Trotter CL, Waight P, Andrews NJ et al. Epidemiology of invasive pneumococcal disease in the pre-conjugate vaccine era: England and Wales, 1996-2006. *J Infect* 2010;60:200-208.
6. Miller E, Andrews NJ, Waight PA *et al*. Herd immunity and serotype replacement 4 years after seven-valent pneumococcal conjugate vaccination in England and Wales: an observational cohort study. *Lancet Infect Dis* 2011; **11**: 760-8.
7. Van Hoek AJ, Andrews N, Waight PA et al. effect of serotype on focus and mortality of invasive pneumococcal disease: coverage of difference vaccines and insight into non-vaccine serotypes. *Plos One*2012; 7(7)e39150.
8. Andrews NJ, Waight PA, George RC *et al*. Impact and effectiveness of 23-valent pneumococcal polysaccharide vaccine against invasive pneumococcal disease in the elderly in England and Wales. *Vaccine* 2012; **30**: 6802-8.
9. Pichon B, Ladhani SN, Slack MP et al. Changes in the molecular epidemiology of streptococcus pneumoniae causing meningitis following introduction of pneumococcal conjugate vaccination in England and Wales. *J Clin Microbiol* 2013;51(3):820-7.
10. Waight PA, Andrews NJ, Ladhani SN *et al*. Effect of the 13-valent pneumococcal conjugate vaccine on invasive pneumococcal disease in England and Wales 4 years after its introduction: an observational cohort study. *Lancet Infect Dis* 2015; **15**: 535-43.
11. Urwin G, Yuan MF, Hall LM et al. Pneumococcal meningitis in the North East Thames Region UK: epidemiology and molecular analysis of isolates. *Epidemiol Infect* 1996; 117(1):95-102.
12. Balakrishnan I, Crook P, Morris R, Gillespie SH. Early predictors of mortality in pneumococcal bacteremia. *J Infect* 2000; 40:256-261.
13. Birtles A, Birgincar N, Sheppard CL et al. Antimicrobial resistance of invasive Streptococcus pneumoniae isolates in a British district general hospital. *J Med Microbiol*2004;53(pt 12):1241-6.
14. Leeming JP, Cartwright K, Morris R et al. Diagnosis of invasive pneumococcal infection by serotype specific urinary antigen detection. *J Clin Microbiol*2005;43(10):4972-4976.
15. Sheppard CL, Salmon JE, Harrison TG et al. The clinical and public health value of non-culture methods in the investigation of a cluster of unexplained pneumonia cases. *Epidemiol Infect* 2008;136:922-927.
16. Ihekweazu CA, Dance DAB, Pebody R et al. Trends in the incidence of pneumococcal disease before introduction of conjugate vaccine: South West England, 1996-2005. *Epidemiol Infect* 2008;136(8):1096-1102.
17. Foster D, Knox K, Walker AS *et al*. Invasive pneumococcal disease: epidemiology in children and adults prior to implementation of the conjugate vaccine in the Oxfordshire region, England. *J Med Microbiol* 2008; **57**: 480-7.
18. Elston JW, Santaniello-Newton A, Meigh JA *et al*. Increasing incidence of invasive pneumococcal disease and pneumonia despite improved vaccination uptake: surveillance in Hull and East Yorkshire, UK, 2002-2009. *Epidemiol Infect* 2012; **140**: 1252-66.
19. Chapman KE, Wilson D, Gorton R. Serotype dynamics of invasive pneumococcal disease post-PCV7 and pre-PCV13 introduction in North East England. *Epidemiol Infect* 2013; **141**: 344-52.
20. Bewick T, Sheppard C, Greenwood S et al. Serotype prevalence in adults hospitalised with pneumococcal non-invasive community-acquired pneumonia. *Thorax* 2012; 67:540-545.
21. Rodrigo C, Bewick T, Sheppard C *et al*. Pneumococcal serotypes in adult non-invasive and invasive pneumonia in relation to child contact and child vaccination status. *Thorax* 2014; **69**: 168-73.
22. Rodrigo C, Bewick T, Sheppard C *et al*. Clinical features of adults with seven-valent-conjugated-vaccine-serotype pneumococcal pneumonia. *Vaccine* 2014; **32**: 1460-5.
23. Rodrigo C, Bewick T, Sheppard C *et al*. Impact of infant 13-valent pneumococcal conjugate vaccine on serotypes in adult pneumonia. *Eur Respir J* 2015; **45**: 1632-41.
24. Kyaw MH, Clarke S, Edwards GF *et al*. Serotypes/groups distribution and antimicrobial resistance of invasive pneumococcal isolates: implications for vaccine strategies. *Epidemiol Infect* 2000; **125**: 561-72.
25. McKenzie H, Reid N< Dijkhuzen RS. Clinical and microbiological epidemiology of Streptococcus pneumoniae bacteraemia. *J Med Microbiol* 2000;49(4):361-6.
26. Kyaw MH, Clarke S, Jones IG *et al*. Non-invasive pneumococcal disease and antimicrobial resistance: vaccine implications. *Epidemiol Infect* 2002; **128**: 21-7.
27. Denham BC, Clarke SC. Serotype incidence and antibiotic susceptibility of Streptococcus pneumoniae causing invasive disease in Scotland, 1999-2002. *J Med Microbiol* 2005; 54(Pt4):327-31.
28. Clarke SC, Scott KJ, McChlery SM. Serotypes and sequence types of pneumococci causing invasive disease in Scotland prior to the introduction of pneumococcal conjugate polysaccharide vaccines. *J Clin Microbiol* 2004; **42**: 4449-52.
29. Mooney JD, Weir A, McMenamin J *et al*. The impact and effectiveness of pneumococcal vaccination in Scotland for those aged 65 and over during winter 2003/2004. *BMC Infect Dis* 2008; **8**: 53.
30. Jeffries JM, Smith AJ, Edwards GF et al. Temporal analysis of invasive pneumococcal clones from Scotland illustrates fluctuations in diversity of serotype and genotype in the absence of pneumococcal conjugate vaccine. *J Clin Microbiol* 2010;48(1):87-96.
31. Inverarity D, Lamb K, Diggle M *et al*. Death or survival from invasive pneumococcal disease in Scotland: associations with serogroups and multilocus sequence types. *J Med Microbiol* 2011; **60**: 793-802.
32. Lamb KE, Flasche S, Diggle M *et al*. Trends in serotypes and sequence types among cases of invasive pneumococcal disease in Scotland, 1999-2010. *Vaccine* 2014; **32**: 4356-63.
33. Henriques B, Kalin M, Ortqvist A et al. Molecular epidemiology of Streptococcus pneumoniae causing invasive disease in 5 countries. *J Infect Dis* 2000;182:833-9.
34. Farrell DJ, Felmingham D, Shackcloth J *et a*l. Non-susceptibility trends and serotype distributions among Streptococcus pneumoniae from community-acquired respiratory tract infections and from bacteraemias in the UK and Ireland, 1999 to 2007. *J Antimicrob Chemother* 2008; **62 Suppl 2**: ii87-95.
35. Feikin DR, Kagucia EW, Loo JD *et al*. Serotype-specific changes in invasive pneumococcal disease after pneumococcal conjugate vaccine introduction: a pooled analysis of multiple surveillance sites. *PLoS Med* 2013; **10**: e1001517.
36. Torne AN, Dias JG, Quinten C et al. European enhanced surveillance of invasive pneumococcal disease in 2010; data from 26 European countries in the post-heptavalent conjugate vaccine era. *Vaccine* 2014;32:3644-3650.
37. Yin Z, Rice BD, Waight P *et al*. Invasive pneumococcal disease among HIV-positive individuals, 2000-2009. *AIDS* 2012; **26**: 87-94.
38. van Hoek AJ, Andrews N, Waight PA *et al*. The effect of underlying clinical conditions on the risk of developing invasive pneumococcal disease in England. *J Infect* 2012; **65**: 17-24.
